# Associations between visual skills and serve reception performance in elite male volleyball outside hitters

**DOI:** 10.3389/fphys.2026.1846247

**Published:** 2026-06-17

**Authors:** Xiushen Dong, Dongxu Gao, Yuqing Gao, Lei Han, Xinhong Zhou, Congcong Weng, Jiayu Wang, Qiangquan Wang, Youlin Xiao, Qingtao Wang, Yuenian Tang

**Affiliations:** 1 College of Physical Education, Hunan Normal University, Changsha, China; 2 Department of Physical Education, Tsinghua University, Beijing, China; 3 School of Physical Education, Wuhan Sports University, Wuhan, China; 4 Department of Physical Education, Jiaxing University, Jiaxing, China; 5 School of Physical Education, Hubei University, Wuhan, China; 6 College of Physical Education, Ningxia Normal University, Guyuan, China; 7 College of Physical Education, Dalian University, Dalian, China; 8 School of Physical Education, Shandong University of Traditional Chinese Medicine, Jinan, China

**Keywords:** data volley, jump serve, outside hitter, Senaptec, serve reception efficiency, visual skills, volleyball

## Abstract

**Introduction:**

Visual skills are considered important for elite athletic performance, but their association with volleyball-specific performance remains unclear. This study examined the associations between visual skills and match-based serve reception performance in elite male volleyball outside hitters.

**Methods:**

Twenty-four elite male volleyball outside hitters were included. Ten visual skills were assessed using the Senaptec Sensory Station, and season-long serve reception performance was analyzed using the Data Volley system. Perfect serve reception rate (#%), defined as the percentage of receptions rated as perfect out of the total number of individual serve receptions, was used as the match-based performance indicator.

**Results:**

Pearson correlation analysis showed that perfect serve reception rate was strongly associated with eye-hand coordination (r = −0.778, p < 0.001), near/far quickness (r = 0.756, p < 0.001), and reaction time (r = −0.746, p < 0.001). A theory-driven parsimonious regression model suggested that these three variables were independently associated with perfect serve reception rate in this sample.

**Discussion:**

Selected visual skills, particularly eye-hand coordination, near/far quickness, and reaction time, are associated with serve reception performance in elite male volleyball outside hitters. Visual skills assessment may provide supplementary information for understanding individual differences in serve reception performance, although these findings should be interpreted cautiously given the cross-sectional design and small sample size.

## 1 Introduction

Volleyball is a high-speed team sport in which performance is influenced by physical fitness, technical skill, tactical organization, and perceptual-cognitive ability. In modern elite men’s volleyball, matches have become faster and more intense, with shorter rallies and increasing demands on rapid information processing (Sheppard et al., 2009; Drikos and Vagenas, 2011; Marcelino et al., 2012; De Alcaraz et al., 2017). In particular, the jump serve has become a dominant serving strategy. Ball speeds often exceed 100 km/h, leaving receivers only a few hundred milliseconds to judge the ball’s speed, trajectory, spin, and landing point (Palao and Valadés, 2014; Palao et al., 2009; Sheppard et al., 2009; Valhondo et al., 2018). Under these constraints, successful serve reception requires not only technical proficiency but also efficient visual information processing, attention allocation, spatial judgment, and rapid motor response.

Sports vision refers to the ability to receive, process, and use visual information to guide movement performance. It includes multiple components, such as visual clarity, contrast sensitivity, depth perception, visual attention, eye-hand coordination, and reaction time. These abilities support perception-action coupling and allow athletes to detect relevant cues, anticipate events, and execute appropriate motor responses in dynamic sport environments (Laby and Appelbaum, 2021; Lochhead et al., 2024). Previous studies in interactive and interceptive sports have shown that visual and perceptual-cognitive abilities are associated with sport-specific performance indicators in baseball, basketball, ice hockey, and boxing (Reichow et al., 2011; Mangine et al., 2014; Poltavski and Biberdorf, 2015; Burris et al., 2018; Laby et al., 2018, 2019; Lochhead et al., 2024). These findings suggest that visual skills may be relevant to performance in sports requiring rapid perception and action under time pressure.

Serve reception in volleyball is a typical visually guided interceptive task (Paulo et al., 2016). During reception, athletes must quickly identify the serve type, track the ball’s flight, estimate its landing position, and coordinate body movement and platform control within a limited time window. Reception quality also plays an important role in subsequent offensive organization (Paulo et al., 2016, 2018). In the Data Volley system, perfect reception is commonly coded as “#”, indicating a reception that allows the setter to organize the attack with full tactical options. Therefore, serve reception success rate based on perfect receptions can be used as a match-based indicator of reception performance. For outside hitters, who frequently perform serve-reception duties in elite men’s volleyball, stable and high-quality reception is especially important for team offensive efficiency (González-Silva et al., 2020).

Although visual skills have been examined in several interactive and interceptive sports, evidence specific to volleyball serve reception remains limited. Previous volleyball research has mainly focused on technical, tactical, and match-performance indicators, whereas few studies have directly examined whether laboratory-based visual skills are associated with match-based serve reception performance. This gap is particularly important because elite serve reception occurs under extreme time pressure and places high demands on visual information processing. Therefore, clarifying these associations is important for understanding individual differences in serve reception performance and may provide a meaningful basis for future visual assessment and training research.

Therefore, this study aimed to examine the associations between selected visual skills and match-based serve reception success rate in elite male volleyball outside hitters. We hypothesized that specific visual skills, particularly those related to rapid visual information processing and visuomotor coordination, would be associated with serve reception success rate.

## 2 Materials and methods

### 2.1 Participants

Twenty-four elite male volleyball outside hitters aged 17–25 years (age 20.75 ± 2.66 years, training experience 8.38 ± 2.62 years) were recruited from the elite group of the National Men’s Volleyball Super League. The participants were professional male volleyball players from teams across 12 provinces. Only outside hitters were included because this playing position typically undertakes substantial serve-reception responsibilities in elite volleyball, making it appropriate for examining individual differences in serve reception performance. Only male athletes were included because the present study focused on elite male volleyball outside hitters within the same competitive context. This sampling strategy was used to reduce heterogeneity related to sex-specific competition characteristics, training background, and match-performance demands. Inclusion criteria were: (a) at least five years of professional volleyball training; (b) participation in at least five national and five regional competitions; (c) registered athlete status in domestic competitions; (d) normal vision and visual function, with no history of eye disease or corrective treatment; (e) no prior systematic visual skills training; and (f) a minimum of 150 serve receptions during the season. Exclusion criteria included: serious lower-limb injury or concussion during the season, or refusal to complete the visual skills assessment. Participants were recruited using a purposive sampling approach based on playing position, competitive level, and availability of season-long match-performance data. This sampling strategy was selected because the study specifically aimed to examine elite male outside hitters who had sufficient serve-reception exposure during the competitive season. All participants were fully informed of the study’s purpose, procedures, and potential risks, and provided written informed consent. The study protocol was approved by the Ethics Committee of Hunan Normal University (see attachment) and strictly followed the ethical principles of the Declaration of Helsinki (2013 revision).

### 2.2 Assessment of visual skills

Visual skills were assessed using the Senaptec Sensory Station (SS201, Senaptec, USA). The reliability and validity of this system have been supported by previous studies (Erickson et al., 2011; Zynda et al., 2024). Ten visual skill metrics were evaluated: visual clarity, contrast sensitivity, depth perception, near/far quickness, perceptual span, multiple object tracking, reaction time, target capture, eye-hand coordination, and Go/No-Go decision-making. The visual skill assessments were conducted using the Senaptec Sensory Station according to standardized testing procedures. The specific assessment items, testing content, and measurement descriptions are presented in **Table 1**.

**Table 1:** Detailed description of basic sports vision tests.

Test variables	Setup and starting position	Task procedures/test instructions	Outcome measures
Visual clarity VC 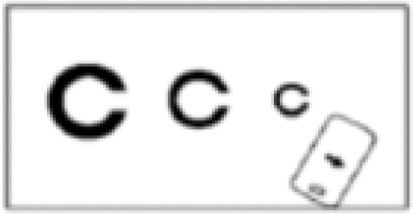	Participants held the mobile device while standing 3 m away from the tablet screen.	Participants judged the direction of the gap in a C-shaped figure displayed on the tablet and swiped in the corresponding direction on the mobile device. Monocular testing was performed first for the left and right eyes, followed by binocular testing	The higher the accuracy of the judgment, the smaller the figure. The test index unit is LogMAR, the smaller the better. In this article, a 5-point conversion method is adopted, that is, 5 minus the score is the final result. This task reflects static visual acuity or the minimum detectable spatial resolution for a non-moving object.
Contrast sensitivity CS 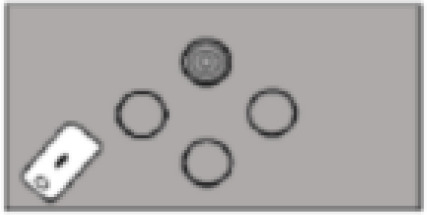		Four black circles appeared on the tablet screen, with one circle randomly containing concentric circles of varying contrast. Participants identified the target circle and swiped in the corresponding direction on the mobile device.	As accuracy increases, the concentric circles’ contrast becomes progressively less visible. Scores are expressed in LogCS, with higher values indicating better performance.
Depth perception DP 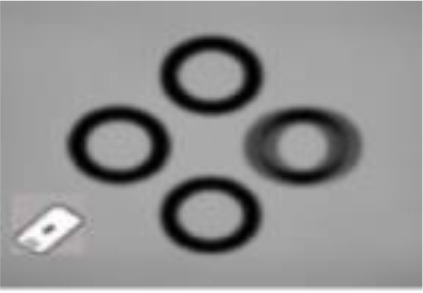		Four black circles appeared on the screen, with one circle randomly displaying a 3D stereoscopic effect. Participants completed binocular assessment first, followed by monocular testing for the right and left eyes.	As accuracy improves, the contrast and stereoscopic effect of the target circle become less pronounced. Scores are expressed in arcseconds (arcsec), with lower values indicating better performance.
Near/far quickness N/F Q 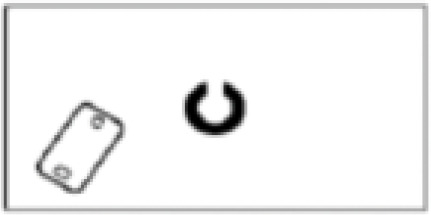	The top of the mobile device was aligned with the bottom of the tablet screen, positioned approximately 40 cm from the participant’s eyes.	C-shaped figures alternately appeared on the Senaptec tablet (far) and mobile device (near). Participants had 30 seconds to switch focus between the far and near screens, judge the gap direction, and swipe the corresponding direction on the mobile device.。	Faster and more accurate gap judgments yield better scores. Performance is assessed by the number of correct swipes within 30 seconds, with higher counts indicating better performance. Reaction times for both near and far screens were measured in milliseconds (ms), with lower values indicating faster responses and better performance.
Perception span PS 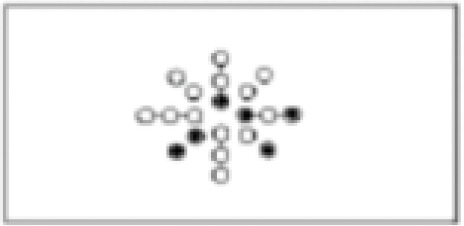	Participants stood 60 cm away from the tablet, with their eyes level with the center of the screen	Four black circles appeared on the screen, with one circle randomly displaying a 3D stereoscopic effect. Participants completed binocular assessment first, followed by monocular testing for the right and left eyes.	The number of circles and black dots increases progressively. Scores are based on the cumulative number of correctly identified targets, with higher counts indicating better performance.
Multiple object tracking MOT 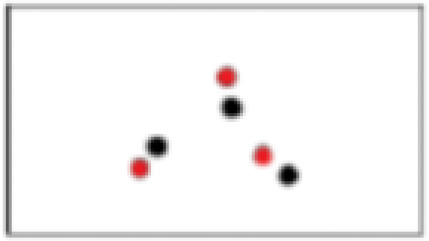		Several pairs of black balls were displayed. In each pair, one ball briefly changed to red before returning to black, then rotated continuously in random clockwise or counterclockwise directions. After the rotation stopped, participants identified the initially red ball in each pair.	Outcome measures include the number of correctly tracked objects, tracking speed (deg/sec), percentage score (%), and a composite score. Higher values indicate better performance.
Reaction time RT 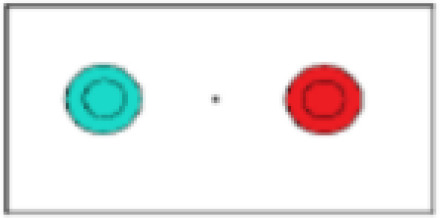		Participants first selected their dominant hand. Two circles appeared on the screen; participants touched each circle with the index fingers of both hands, turning the circles green. The circles then randomly turned red, and participants were instructed to lift and then replace the corresponding finger as quickly as possible.	Includes measurements for dominant hand, non-dominant hand, and average reaction time. Units are in milliseconds (ms), with faster movements indicating better performance.
Target capture TC 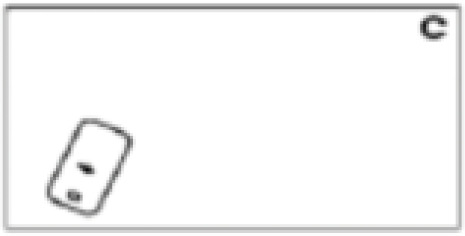	For tests using the large display, participants stood 3 m away, aligning their line of sight with a blue reference line at the center of the screen.	Participants fixated on the center of the large display. C-shaped figures then appeared randomly in the four corners. Participants judged the gap direction and swiped in the corresponding direction on the mobile device.	Scores are measured in milliseconds (ms), with faster responses indicating better inhibitory control.
Eye-hand coordination EHC 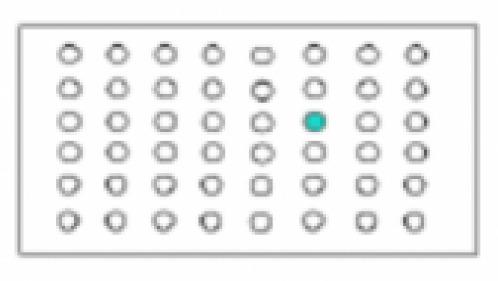	When standing 60 cm from the large display, the screen’s central line was raised to approximately chest or arm level to avoid interference with the participant’s peripheral vision during target touch tasks.	On the large display, 8 columns × 10 rows of hollow circles appeared. During testing, one circle randomly changed to cyan. After the participant tapped it, another circle appeared in a new location. Participants were instructed to tap as many targets as accurately and quickly as possible within the time limit.	Outcome measures include total time, average reaction time, reaction time for central and peripheral regions (ms), with lower values indicating better performance.
Go/no-go G/NG 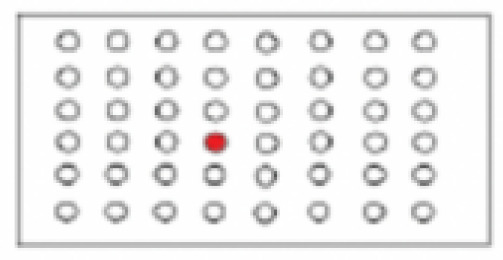		Similar 8-column circles appeared on the large display. Green dots required rapid tapping, whereas red dots required withholding the response. Participants followed the rules to respond correctly to the target stimuli.	Scoring includes overall accuracy: correct taps increase the score, while incorrect taps reduce the score. Higher overall scores indicate better performance, and lower error scores indicate better inhibitory control.

All visual ability tests were conducted in the sport-specific vision laboratory and completed within one week before the season started. For each test, a demonstration and three practice trials were provided prior to the official assessment.

### 2.3 Serve reception performance analysis

Serve reception performance was analyzed using Data Volley 4 Professional (Data Project, Italy) to perform technical and statistical analysis on the season match videos of all participants. This method is widely used in volleyball performance research. Data coding was conducted by trained analysts according to the standardized Data Volley five-level serve reception scoring system. Before formal data collection, the analysts completed a calibration session using 50 serve reception actions to ensure consistency in the interpretation of scoring criteria. Inter-rater reliability between the two independent analysts was assessed using the intraclass correlation coefficient (ICC), and the agreement showed a high level of reliability (ICC = 0.921).Perfect serve reception rate (#%) was selected as the primary performance indicator. It was defined as the percentage of perfect receptions rated “#” in the Data Volley five-level scoring system out of the total number of individual serve receptions during the season, calculated as:


$$\#\%=\frac{Number\;of\;perfect\;receptions}{Total\;number\;of\;individual\;serve\;receptions\;}\times 100.$$

In this system, a “#” rating indicates that the setter gains full tactical options and can organize a first-tempo attack, representing the highest quality of serve reception. This metric has a clear operational definition, does not rely on weighting assumptions, and directly reflects the contribution of serve reception quality to offensive organization (Palao et al., 2004, 2009; Silva et al., 2016). The Data Volley scoring system is presented in **Table 2**.

**Table 2:** Data volley 5-level serve-receive scoring system.

Level	Symbol	Meaning	Impact on offense
Perfect	#	Setter has full offensive options	Any tactic can be executed (e.g., quick attack)
Positive	+	Setter has two main offensive options	Limited but can organize offense
Acceptable	!	Setter has only one option	Offense is clearly restricted
Negative	−	Non-setter forced to organize	Offensive control largely lost
Poor	/	Ball barely in play	Offensive control completely lost

The primary dependent variable in this study is the perfect serve-receive rate (#%). *(References:* Palao et al., 2004; Afonso et al., 2012).

### 2.4 Procedures

The study was conducted in two phases. In the first phase, visual skills were assessed before the start of the season. In the second phase, serve reception performance data were collected throughout the season. After the season, visual test results were matched with performance data for statistical analysis. Prior to formal data collection, ethical approval was obtained, club collaboration agreements were signed, and participants were recruited. Eligible elite outside hitters were selected based on the inclusion and exclusion criteria. Two Data Volley analysts were trained 2–3 weeks before the season, using similar match videos to complete 50 serve reception coding exercises to ensure consistency in scoring standards. One week before the season, participants were briefed on the study purpose and procedures, provided informed consent, and completed a basic information questionnaire.

Visual skills testing was conducted one week before the season, scheduled from 9:00 to 11:00 to minimize the effects of circadian rhythm on reaction time and visual processing. Tests were carried out in the sports vision laboratory using the Senaptec Sensory Station (Senaptec Inc., USA). The assessment included visual clarity, contrast sensitivity, depth perception, near/far quickness, target capture, perceptual span, multiple object tracking, eye-hand coordination, response inhibition (Go/No-Go), and reaction time. Each test was performed following standardized demonstration and practice before recording formal scores.

Performance data were collected during the 2025 season. Matches were recorded using dual high-definition cameras (≥1080p/60 fps), with one positioned at the end line and the other at a 45° side angle, and recordings were time-synchronized. Video archiving was completed within 24 hours after each match. Using Data Volley 4, the two analysts independently scored each serve reception on a five-point scale and recorded the type of serve (jump serve, float jump serve, or other). Using Data Volley 4, serve reception actions were coded according to the standardized five-level scoring system, and the type of serve was recorded as jump serve, float jump serve, or other. All coded data were checked for completeness and consistency before statistical analysis.

### 2.5 Statistical analysis

All statistical analyses were performed using IBM SPSS Statistics 27.0. Descriptive statistics are presented as mean ± standard deviation (M ± SD). The normality of continuous variables was assessed using the Shapiro–Wilk test (normality assumed when p > 0.05). Pearson correlation analysis was used to examine the associations between visual skill metrics and perfect serve reception rate (#%). Correlation coefficients (r), 95% confidence intervals (95% CI), and p values were reported, and the strength of correlations was interpreted according to Cohen’s criteria. To reduce the risk of false-positive findings due to multiple correlation tests, false discovery rate (FDR) correction was additionally applied.

Due to the small sample size, a theory-driven parsimonious multiple linear regression model was constructed to examine whether selected visual skill variables were independently associated with perfect serve reception rate. Predictors were selected based on theoretical relevance, bivariate correlation strength, and the need to limit model complexity. Model assumptions were assessed using residual diagnostics, including residual normality, linearity, homoscedasticity, multicollinearity using variance inflation factors (VIF), and influential observations using Cook’s distance. As the study focused on a highly specific elite-athlete population and the available sample was constrained by playing position, competitive level, and season match exposure, an *a priori* sample size calculation was not used to determine recruitment. Instead, a *post hoc* sensitivity analysis was conducted using G*Power to estimate the minimum detectable effect size under the available sample size. A G*Power sensitivity analysis was conducted to estimate the minimum detectable effect size under the available sample size (Faul et al., 2009).

## 3 Results

### 3.1 Descriptive statistics and normality

Descriptive statistics for the 24 male elite volleyball outside hitters are presented in **Table 3**. Participants had a mean age of 20.75 ± 2.66 years and an average training experience of 8.38 ± 2.62 years. Their mean height and weight were 183.67 ± 5.53 cm and 78.92 ± 11.90 kg, respectively. For visual skill variables, the mean values were −0.07 ± 0.08 logMAR for VC, 1.73 ± 0.28 logCS for CS, 150.83 ± 52.79 arcsec for DP, 15.79 ± 4.25 for NFQ, 296.83 ± 68.95 ms for TC, 42.83 ± 7.39 for PS, 0.78 ± 0.12 for MOT, 51.73 ± 4.03 s/trial for EHC, 5.62 ± 1.21 for GNG, and 302.50 ± 29.95 ms for RT. The mean perfect serve reception rate (#%) was 26.29 ± 6.51%(This rate indicates that about one quarter of serve receptions were perfect, representing an expected and practically meaningful level for elite outside hitters in high-level men’s volleyball).The inter-rater reliability for serve reception scoring showed a high level of reliability (ICC = 0.921), indicating good consistency between the two independent analysts.

**Table 3:** Descriptive statistics and normality test results.

Parameter	Unit	M ± SD (n=24)	Minimum	Maximum	Shapiro-W	Shapiro-p
Age	y	20.75 ± 2.66	17	25	0.929	0.0915
Height	cm	183.67 ± 5.53	170	194	0.970	0.679
Weight	kg	78.92 ± 11.90	55	95	0.930	0.0984
Experience	y	8.38 ± 2.62	5	14	0.944	0.2031
VC	logMAR-	−0.07 ± 0.08	−0.22	0.10	0.985	0.9686
CS	logCS+	1.73 ± 0.28	1.29	2.40	0.953	0.3159
DP	arcsec-	150.83 ± 52.79	71	284	0.963	0.494
NFQ	score+	15.79 ± 4.25	7	23	0.975	0.7787
TC	ms-	296.83 ± 68.95	173	429	0.976	0.8175
PS	score+	42.83 ± 7.39	31	58	0.970	0.6765
MOT	score+	0.78 ± 0.12	0.56	0.96	0.957	0.3818
EHC	s-	51.73 ± 4.03	44.4	59.78	0.974	0.7616
GNG	score+	5.62 ± 1.21	3	8	0.925	0.0769
RT	ms-	302.50 ± 29.95	241	364	0.998	1.000
#%	%	26.29 ± 6.51	14.5	36.4	0.953	0.3207

M, mean; SD, standard deviation; Shapiro–W, Shapiro–Wilk statistic. A superscript “+” indicates that higher scores reflect better performance, while a superscript “−” indicates that lower values reflect better performance.

The Shapiro–Wilk test showed that all variables were approximately normally distributed, with p values greater than 0.05. Specifically, the normality test results ranged from W = 0.925 to 0.998, with p values ranging from 0.077 to 1.000. These results indicated no significant deviation from normality for demographic variables, visual skill metrics, or perfect serve reception rate, supporting the use of Pearson correlation analysis and linear regression in subsequent analyses.

### 3.2 Pearson correlation analysis

**Figure 1:**
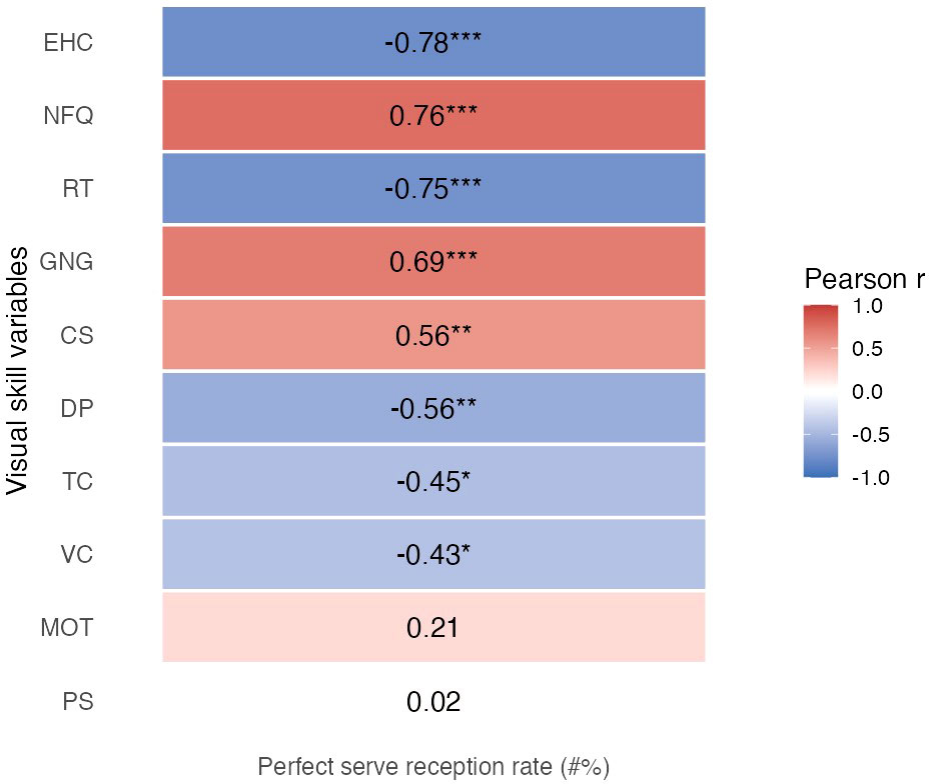
Heatmap of Pearson correlations between visual skill variables and perfect serve reception rate (#%). Values inside the heatmap represent Pearson correlation coefficients between visual skill variables and perfect serve reception rate (#%). Statistical significance was determined after false discovery rate (FDR) correction using the Benjamini–Hochberg method. *p< 0.05, **p< 0.01, ***p< 0.001 after FDR correction. Positive coefficients indicate that higher visual skill scores were associated with higher perfect serve reception rates, whereas negative coefficients indicate that lower values of the corresponding visual skill measures were associated with higher perfect serve reception rates.

Pearson correlation analysis between visual skill variables and perfect serve reception rate (#%) is presented in **Table 4** and visually summarized in **Figure 1**. After FDR correction, significant associations remained for eight visual skill variables. Based on Cohen’s effect size classification, very strong correlations were observed for eye–hand coordination (EHC, r = −0.778, 95% CI: −0.899 to −0.546, p< 0.001, FDR-adjusted p< 0.001), near/far quickness (NFQ, r = 0.756, 95% CI: 0.508 to 0.889, p< 0.001, FDR-adjusted p< 0.001), and reaction time (RT, r = −0.746, 95% CI: −0.883 to −0.490, p< 0.001, FDR-adjusted p< 0.001). Strong correlations were found for Go/No-Go (GNG, r = 0.688, 95% CI: 0.393 to 0.854, p< 0.001, FDR-adjusted p< 0.001), contrast sensitivity (CS, r = 0.560, 95% CI: 0.203 to 0.786, p = 0.004, FDR-adjusted p = 0.008), and depth perception (DP, r = −0.557, 95% CI: −0.784 to −0.198, p = 0.005, FDR-adjusted p = 0.008). Moderate significant correlations were observed for target capture (TC, r = −0.447, 95% CI: −0.720 to −0.053, p = 0.029, FDR-adjusted p = 0.041) and visual clarity (VC, r = −0.425, 95% CI: −0.707 to −0.027, p = 0.038, FDR-adjusted p = 0.048). Multiple object tracking (MOT, r = 0.210, 95% CI: −0.212 to 0.565, p = 0.325, FDR-adjusted p = 0.361) and perceptual span (PS, r = 0.016, 95% CI: −0.390 to 0.417, p = 0.941, FDR-adjusted p = 0.941) were not significantly associated with perfect serve reception rate.

**Table 4:** Pearson correlation between visual ability variables and perfect serve-receive rate (#%) (n = 24).

Variable	Unit	r	p	Effect	95% CI	FDR-adjusted p
EHC	s	-0.778	<0.001	Very strong	-0.899 to -0.546	<0.001
NFQ	score	0.756	<0.001	Very strong	0.508 to 0.889	<0.001
RT	ms	-0.746	<0.001	Very strong	-0.883 to -0.490	<0.001
GNG	score	0.688	<0.001	Strong	0.393 to 0.854	<0.001
CS	logCS	0.560	0.004	Strong	0.203 to 0.786	0.008
DP	arcsec	-0.557	0.005	Strong	-0.784 to -0.198	0.008
TC	ms	-0.447	0.029	Moderate	-0.720 to -0.053	0.041
VC	logMAR	-0.425	0.038	Moderate	-0.707 to -0.027	0.048
MOT	ratio	0.210	0.325	Weak	-0.212 to 0.565	0.361
PS	score	0.016	0.941	Weak	-0.390 to 0.417	0.941

r, Pearson correlation coefficient; 95% CI, 95% confidence interval; FDR-adjusted p values were calculated using the Benjamini–Hochberg method. Effect size classification was based on Cohen’s criteria: |r|< 0.30 = weak, 0.30 ≤ |r|< 0.50 = moderate, 0.50 ≤ |r|< 0.70 = strong, and |r| ≥ 0.70 = very strong.

**Figure 1** further illustrates the direction and magnitude of these associations. Negative correlations were observed for EHC, RT, DP, TC, and VC, indicating that lower values in these measures were associated with higher perfect serve reception rates, which is consistent with the scoring direction of these tests. Positive correlations were observed for NFQ, GNG, CS, MOT, and PS. The strongest associations were visually concentrated in EHC, NFQ, and RT, supporting their inclusion in the subsequent parsimonious regression model. Overall, these findings indicate that several visual skill variables, particularly EHC, NFQ, and RT, were strongly associated with serve reception performance.

### 3.3 Regression analysis

A theory-driven parsimonious multiple linear regression model was constructed using RT, EHC, and NFQ as predictors of perfect serve reception rate (#%), with the results presented in **Table 5**. The overall model was statistically significant, F(3, 20) = 28.009, p< 0.001, and accounted for 80.8% of the variance in perfect serve reception rate in this sample, with an adjusted R² of 0.779. All three predictors were significantly associated with perfect serve reception rate. EHC showed the strongest standardized association, B = −0.648, SE = 0.207, β = −0.401, 95% CI: −1.079 to −0.217, t = −3.13, p = 0.005. NFQ was positively associated with perfect serve reception rate, B = 0.529, SE = 0.197, β = 0.346, 95% CI: 0.118 to 0.940, t = 2.68, p = 0.014. RT was negatively associated with perfect serve reception rate, B = −0.068, SE = 0.028, β = −0.314, 95% CI: −0.127 to −0.010, t = −2.43, p = 0.025. The VIF values ranged from 1.70 to 1.74, indicating no substantial multicollinearity among the predictors.

**Table 5:** Parsimonious multiple linear regression model predicting perfect serve reception rate.

Predictor	B	SE	*β*	95% CI for B	t	p	VIF
RT	-0.068	0.028	-0.314	-0.127 to -0.010	-2.43	0.025	1.70
EHC	-0.648	0.207	-0.401	-1.079 to -0.217	-3.13	0.005	1.74
NFQ	0.529	0.197	0.346	0.118 to 0.940	2.68	0.014	1.70
**Model fit:** R² = 0.808; Adjusted R² = 0.779; F(3, 20) = 28.009; p< 0.001.							

RT, reaction time; EHC, eye–hand coordination; NFQ, near/far quickness; B, unstandardized regression coefficient; SE, standard error; β, standardized regression coefficient; CI, confidence interval; VIF, variance inflation factor. Bold values indicate statistically significant results at p < 0.05.

Model diagnostic results are presented in **Table 6**. The residuals showed no significant deviation from normality according to the Shapiro–Wilk test, W = 0.986, p = 0.973. The Breusch–Pagan test showed no evidence of heteroscedasticity, χ² = 6.44, df = 3, p = 0.092, and the Durbin–Watson test indicated no evidence of residual autocorrelation, DW = 1.43, p = 0.176. Cook’s distance analysis is presented in **Table 7**. The maximum Cook’s distance was 0.418, and three observations exceeded the conventional 4/n threshold of 0.167. However, no observation exceeded Cook’s distance of 1, suggesting that no extreme influential observation was identified. Given the small sample size, the regression findings should still be interpreted cautiously as exploratory evidence.

**Table 6:** Diagnostic tests for the parsimonious multiple linear regression model.

Assumption	Test	Statistic	df	p	Interpretation
Residual normality	Shapiro–Wilk test	W = 0.986	—	0.973	No evidence of non-normal residuals
Homoscedasticity	Breusch–Pagan test	χ² = 6.44	3	0.092	No evidence of heteroscedasticity
Independence of residuals	Durbin–Watson test	DW = 1.43	—	0.176	No evidence of autocorrelation

**Table 7:** Cook’s distance analysis for influential observations.

Indicator	Value
Maximum Cook’s distance	0.418
Threshold based on 4/n	0.167
Number of observations above 4/n	3
Number of observations above 1	0

**Figure 2:**
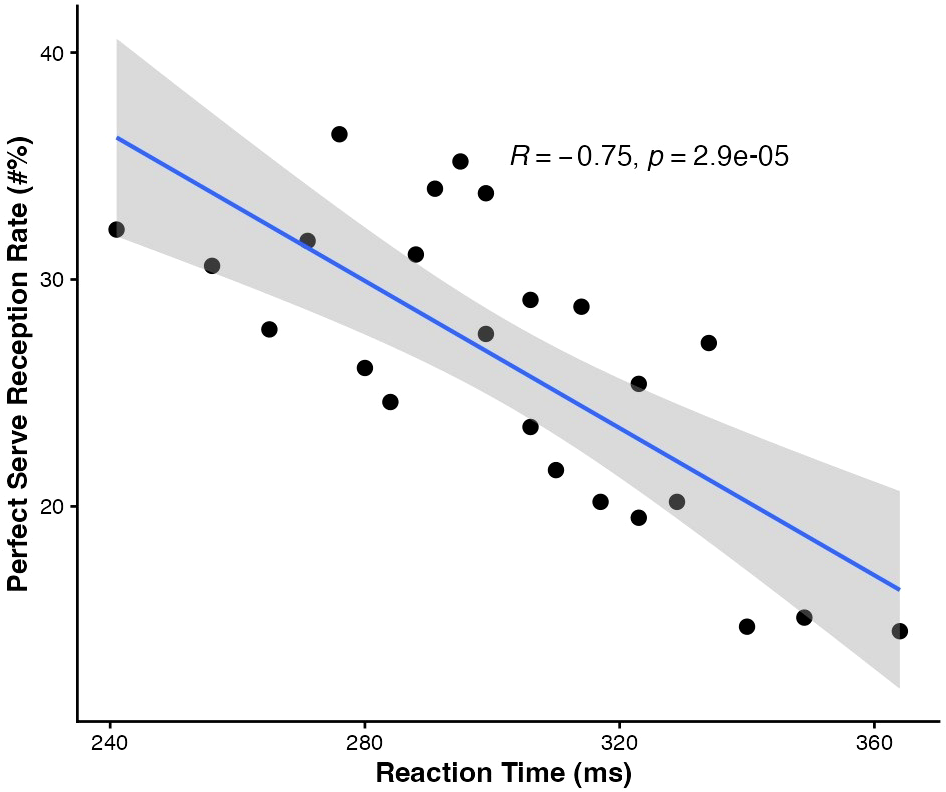
Relationship between reaction time (RT) and perfect serve reception rate (#%). The solid line represents the fitted linear regression line, and the shaded area indicates the 95% confidence interval. R represents Pearson’s correlation coefficient, and the p value indicates the statistical significance of the correlation.

**Figure 3:**
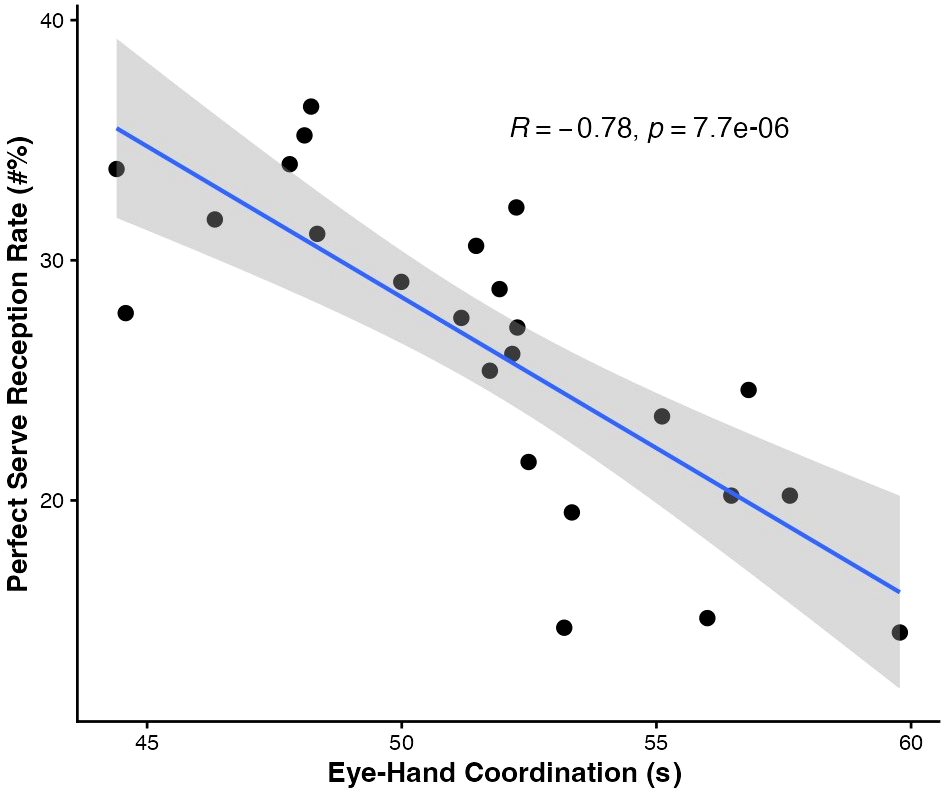
Relationship between eye–hand coordination (EHC) and perfect serve reception rate (#%). The solid line represents the fitted linear regression line, and the shaded area indicates the 95% confidence interval. R represents Pearson’s correlation coefficient, and the p value indicates the statistical significance of the correlation.

**Figure 4:**
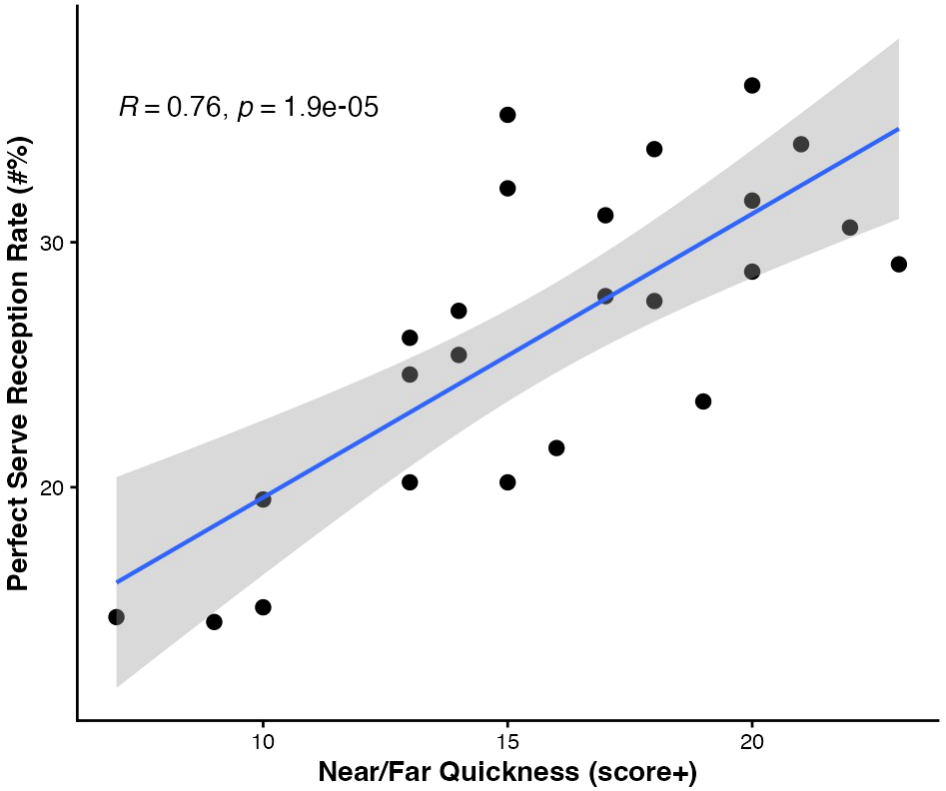
Relationship between near/far quickness (NFQ) and perfect serve reception rate (#%). The solid line represents the fitted linear regression line, and the shaded area indicates the 95% confidence interval. R represents Pearson’s correlation coefficient, and the p value indicates the statistical significance of the correlation.

To visually illustrate the bivariate relationships between the three selected visual skill variables and perfect serve reception rate (#%), **Figures 2**–**4** present scatter plots with fitted regression lines for RT, EHC, and NFQ, respectively. The plots show negative associations for RT and EHC and a positive association for NFQ, consistent with the regression results.

### 3.4 Sensitivity analysis

A sensitivity analysis was conducted to examine whether the regression results were driven by potentially influential observations. Based on Cook’s distance, three observations exceeded the conventional 4/n threshold and were therefore excluded in the sensitivity analysis. After excluding these observations, the parsimonious regression model including RT, EHC, and NFQ remained statistically significant, F(3, 17) = 36.46, p< 0.001, with R² = 0.865 and adjusted R² = 0.842.

Among the three predictors, EHC remained significantly associated with perfect serve reception rate, B = −0.961, 95% CI: −1.367 to −0.554, p< 0.001. RT showed a marginal negative association, B = −0.061, 95% CI: −0.125 to 0.002, p = 0.059, whereas NFQ was no longer statistically significant, B = 0.297, 95% CI: −0.118 to 0.712, p = 0.150. These findings suggest that the overall model fit was not driven by a single extreme influential case. However, the attenuation of the independent associations for RT and NFQ after excluding potentially influential observations indicates that these effects should be interpreted cautiously. Given the small sample size, the regression findings should be considered exploratory and require confirmation in larger independent samples. The results of the sensitivity analysis are presented in **Tables 8** and **9**.

**Table 8:** Sensitivity analysis after excluding potentially influential observations.

Predictor	B	SE	95% CI for B	t	p
RT	-0.061	0.030	-0.125 to 0.002	-2.03	0.059
EHC	-0.961	0.193	-1.367 to -0.554	-4.98	<0.001
NFQ	0.297	0.197	-0.118 to 0.712	1.51	0.150
**Model fit:** R² = 0.865; Adjusted R² = 0.842; F(3, 17) = 36.46; p< 0.001; residual standard error = 2.519; n = 21.					

B, unstandardized regression coefficient; SE, standard error; CI, confidence interval. Sensitivity analysis was performed after excluding three observations with Cook’s distance values greater than the conventional 4/n threshold. The dependent variable was perfect serve reception rate (#%). Bold values indicate statistically significant results at p < 0.05.

**Table 9:** Sensitivity analysis and observed effect size for the parsimonious regression model.

Analysis	n	Predictors	*α*	Target power	df1	df2	f²	R²
Sensitivity analysis for multiple linear regression	24	3	0.05	0.80	3	20	0.553	0.356
Observed model effect size	24	3	—	—	3	20	4.201	0.808

The sensitivity analysis was conducted using G*Power 3.1 for a multiple linear regression model with three predictors, *α* = 0.05, and target power = 0.80. Under these conditions, the minimum detectable effect size was Cohen’s f² = 0.553, corresponding to R² = 0.356. The observed effect size was calculated from the fitted parsimonious regression model, in which R² = 0.808 and Cohen’s f² = 4.201. Cohen’s f² was calculated as R²/(1 − R²).

## 4 Discussion

The present study examined the associations between visual skills and match-based serve reception performance in elite male volleyball outside hitters. The results showed that RT, EHC, and NFQ were very strongly associated with serve reception success rate. GNG, DP, and CS showed strong associations, whereas TC and VC showed moderate associations. No significant associations were observed for MOT and PS. Multiple linear regression analysis further suggested that RT, EHC, and NFQ were independently associated with serve reception success rate in this sample. The lack of significant associations for MOT and PS may be related to differences in task characteristics and visual demands across sports (Nascimento et al., 2020). These findings are generally consistent with previous studies reporting associations between visual skills and athletic performance (Liu et al., 2020; Frolova et al., 2021; Trecroci et al., 2021). However, because this study used a cross-sectional correlational design, these findings should be interpreted as associations rather than evidence of causal effects.

The relationship between visual skills and athletic performance can be interpreted through the sport information processing model (Burris et al., 2018). This model proposes that motor behavior involves perceptual, decision-making, and action-execution processes (Welford, 1960; Adams and Welford, 1970). In volleyball serve reception, athletes must rapidly extract key visual information about ball trajectory and spatial position. They then need to select an appropriate response and adjust their movement when necessary, which may involve inhibitory control mechanisms (Verbruggen and Logan, 2008a, b). Finally, the selected action must be executed accurately to complete the reception. From this perspective, visual processing skills may be associated with serve reception performance through their links with information filtering, response inhibition, and visuomotor execution. Previous studies have also reported associations between visual skills and athletic performance across several sports (Molia et al., 1998; Mangine et al., 2014; Poltavski and Biberdorf, 2015; Burris et al., 2018; Laby et al., 2019; Burris et al., 2020; Liu et al., 2020). Overall, the sport information processing model provides a useful framework for understanding why specific visual skills may be relevant to volleyball serve reception performance.

RT is commonly regarded as an indicator of perceptual–motor conversion efficiency and information processing speed. In this study, RT was strongly associated with outside hitters’ serve reception performance, consistent with findings in ice hockey players (Poltavski and Biberdorf, 2015). In elite volleyball, jump serves are characterized by substantial temporal pressure, providing receivers with only a limited time window from ball release to arrival in the reception zone (Benerink et al., 2015; Paulo et al., 2016). Therefore, serve reception players must capture visual information and prepare motor responses within a very short timeframe. RT may therefore be particularly relevant in this highly reactive and time-constrained task. In contrast, reaction time has shown no significant correlation with performance measures in basketball players (Mangine et al., 2014). This difference may be explained by task structure: basketball offense and defense often involve more proactive decision-making and greater temporal flexibility, whereas volleyball serve reception requires immediate responses to an externally paced stimulus (Paulo et al., 2016, 2018). Thus, the association between RT and performance may depend on the temporal demands of the specific sport task.

EHC reflects the integration of visual input with motor output (Vickers, 2007; Williams and Ford, 2008). Previous studies have shown that elite athletes often perform better than non-athletes in visuomotor integration tasks, and this ability is associated with competitive level (Mann et al., 2007; Appelbaum and Erickson, 2018). During volleyball serve reception, athletes must execute precise arm contact while continuously tracking the ball visually (Sheppard et al., 2009). This process requires coordinated body positioning, platform control, and contact timing. Therefore, the significant association between EHC and serve reception performance is consistent with the perception–action coupling demands of volleyball. In addition, EHC appeared to be the most stable variable in the sensitivity analysis, suggesting that its association with serve reception performance may be more robust than those of RT and NFQ in the present sample.

The present study also found a strong positive correlation between NFQ and serve reception performance. NFQ reflects the ability to rapidly adjust visual focus between near and far targets. In serve reception, athletes must shift attention from the distant server’s movement cues to the approaching ball and then adjust visual focus as the ball nears the body. In addition, this association may also be partly explained by predictive saccadic behavior. In interceptive sports, skilled performers can extract information from the early phase of ball flight and then generate saccades toward the anticipated interception location (Mann et al., 2007). In volleyball serve reception, receivers may similarly use early server and ball-flight cues to predict the reception zone and rapidly shift gaze toward the expected contact area. Therefore, better NFQ may support this rapid far-to-near gaze transition and contribute to more accurate serve reception. This continuous near-to-far visual adjustment places high demands on accommodation and convergence control. Previous research has shown that accommodative flexibility is associated with interception performance (Gao et al., 2015), and volleyball players may show superior accommodative abilities compared with non-athletes (Jafarzadehpur et al., 2007). Therefore, stronger NFQ ability may be related to more efficient visual adjustment during serve reception. Nevertheless, because the sensitivity analysis showed that the independent association of NFQ was attenuated after excluding potentially influential observations, this finding should be interpreted cautiously and requires confirmation in larger samples.

This study found that GNG, DP, and CS were strongly correlated with serve reception performance. First, GNG reflects response inhibition and decision control (Verbruggen and Logan, 2008a, b). During serve reception, when the ball’s trajectory changes or an initial prediction is incorrect, athletes may need to inhibit the original movement plan and adjust their response. Thus, inhibitory control may be relevant to reception performance in this context. Second, DP reflects three-dimensional spatial awareness of distances and relative positions (Howard and Rogers, 2012). Previous studies have shown that depth judgment is related to spatial orientation, action timing, and target-location prediction (Williams et al., 2000; Mann et al., 2007). In serve reception, athletes must judge the flight distance and landing point of the ball to select the appropriate movement direction, step length, and contact timing (Sheppard et al., 2009). Superior depth perception may therefore be associated with more accurate landing-point estimation and movement adjustment. Finally, CS reflects the ability to detect targets under varying contrast conditions and is an important early-stage measure of visual information processing (Ciuffreda and Wang, 2004). Its relationship with athletic performance may vary across sports. For example, contrast sensitivity is significantly associated with competitive level in baseball players (Laby et al., 2018, 2019), but shows weaker associations in ice hockey (Poltavski and Biberdorf, 2015). In volleyball serve reception, the ball is fast, may be affected by lighting and background conditions, and often involves spin and trajectory deviations. Previous research indicates that ball flight trajectory is influenced by speed, spin, and aerodynamic properties, increasing the difficulty of trajectory judgment during reception (Paulo et al., 2016; Tamaru et al., 2020). Therefore, higher CS may be related to more efficient early ball recognition and trajectory information pickup. However, because GNG, DP, and CS were not retained as independent variables in the parsimonious regression model, their roles should be interpreted as task-relevant associations rather than independent explanatory factors.

In this study, TC and VC showed moderate significant correlations with serve reception success rate, but their independent associations appeared to be relatively limited. TC primarily reflects the ability to rapidly shift attention between central and peripheral visual fields, whereas VC represents static visual acuity. In the multiple regression analysis, neither VC nor TC emerged as a significant independent variable associated with serve reception success rate, suggesting limited independent explanatory contribution in this sample. Previous research suggests that basic visual functions in elite athletes are typically high and relatively stable, offering limited discriminative value for performance, whereas higher-level visual processing abilities, such as RT and EHC, may show stronger associations with sport-specific performance (Ciuffreda and Wang, 2004; Hoshina et al., 2013). Considering the characteristics of volleyball serve reception, athletes primarily track a single incoming ball, with relatively limited demand for peripheral target capture. Moreover, elite athletes generally have near-optimal visual acuity, with small individual differences; in this study, both laboratory tests and match contexts used corrected vision when needed. Related studies also indicate that, after vision correction, static visual acuity has a reduced influence on dynamic or sport-specific visual measures (Lee et al., 2022). Therefore, while VC and TC may provide foundational visual support, their independent association with serve reception performance appears to be limited in this sample.

In this study, MOT and PS were not significantly correlated with serve reception success rate. This result may reflect the visual demand structure of volleyball serve reception. Compared with sports such as soccer or ice hockey, which require simultaneous processing of multiple dynamic targets, volleyball serve reception primarily focuses on a single moving ball. Consequently, the role of MOT in this context may be relatively limited. PS in the Senaptec system mainly reflects the ability to recognize and recall visual patterns. However, during volleyball serve reception, athletes’ visual attention is mainly directed toward the incoming ball, body positioning, and immediate contact preparation, with less demand for broader visual pattern processing. Therefore, the lack of significant association between PS and serve reception success rate may indicate that this ability plays a limited role in this specific task.

Although the regression model showed a high explanatory value, this finding should be interpreted cautiously. The sample size was relatively small, and the participants were drawn from a highly specific elite-athlete population. Therefore, the high R² value may partly reflect sample characteristics and potential model instability. The sensitivity analysis showed that after excluding potentially influential observations, the overall model remained significant, but the independent associations for RT and NFQ were attenuated. This suggests that EHC may be the most stable variable in the current model, whereas the independent associations of RT and NFQ require confirmation in larger independent samples. Accordingly, the regression findings should be regarded as exploratory rather than confirmatory evidence.

Previous research has demonstrated associations between visual skills and athletic performance in sports such as baseball, basketball, and ice hockey (Poltavski and Biberdorf, 2015; Burris et al., 2018; Laby et al., 2019; Burris et al., 2020). The present study provides additional evidence in the context of volleyball serve reception. However, the results also suggest that the association between visual skills and performance may not be uniform across sports but may depend on task-specific demands. This interpretation is consistent with the view that differences in task characteristics, environmental constraints, and perception–action coupling determine the relative relevance of specific visual abilities across sports (Nascimento et al., 2020).

From a practical perspective, the present findings highlight the potential importance of reaction time, eye–hand coordination, and near/far quickness in elite serve reception. Coaches may use these indicators to evaluate athletes’ visual profiles, identify individual weaknesses, and provide objective reference information for talent identification and individualized training. Practitioners may also integrate visual training into regular physical and technical training, such as stroboscopic or unpredictable stimulus-response drills for reaction time, reaction-light or ball-tracking tasks for eye–hand coordination, and near–far switching tasks for near/far quickness. However, because this study was correlational, these suggestions should be interpreted as practical directions rather than direct evidence that visual training improves match-based serve reception performance.

## 5 Limitations

This study has several limitations. First, the cross-sectional correlational design only allows for identification of associations between visual skills and serve reception performance, and does not permit causal inferences. Visual abilities were assessed only before the start of the season and therefore may not fully reflect athletes’ visual skills during or after the competitive season. Future studies should conduct repeated visual assessments during or after the season. Second, although the standardized visual assessments used here are highly practical, they differ from the complex perception–action coupling situations in real matches, limiting ecological validity. Additionally, the sample consisted exclusively of elite male volleyball outside hitters aged 17–25 years. While this provides a focused and sport-specific sample, the sample size was relatively small, which may have limited statistical power to detect some potential relationships and restricts the generalizability of the findings. Moreover, because only male athletes were included, the present findings may not be directly generalizable to female volleyball players. The restricted age range also limits the applicability of the results to younger athletes, older professional players, or athletes at different developmental stages. Finally, match performance is influenced by multiple factors, including technique, tactics, experience, and situational context. Future studies should combine more sport-specific task designs with comprehensive performance variables to further clarify how visual skills are associated with volleyball-specific performance.

## 6 Conclusion

This study provides preliminary evidence that selected visual skills are associated with match-based serve reception performance in elite male volleyball outside hitters. Reaction time, eye-hand coordination, and near/far quickness appeared to be the visual skill factors most strongly associated with serve reception success rate in this sample. These findings suggest that high-level serve reception may be related not only to basic visual function but also to rapid information processing, visual accommodation, and visuomotor coordination. Visual skill assessment may therefore provide supplementary information for understanding individual differences in serve reception performance. However, given the cross-sectional correlational design, small sample size, and male-only outside-hitter sample, these findings should be interpreted cautiously. Future studies should further examine different match tasks, playing positions, and intervention designs to better understand how visual skills are associated with competitive performance, thereby providing more robust evidence for elite volleyball training and talent identification.

## Data Availability

The original contributions presented in the study are included in the article/supplementary material. Further inquiries can be directed to the corresponding authors.
